# Multivariate classification of drug-naive obsessive-compulsive disorder patients and healthy controls by applying an SVM to resting-state functional MRI data

**DOI:** 10.1186/s12888-019-2184-6

**Published:** 2019-07-05

**Authors:** Xi Yang, Xinyu Hu, Wanjie Tang, Bin Li, Yanchun Yang, Qiyong Gong, Xiaoqi Huang

**Affiliations:** 1 Mental Health Center Department of Psychiatry, West China Hospital Sichuan University, Chengdu, China; 2 Shenzhen Mental Health Center, Shenzhen, China; 3 Huaxi MR Research Center (HMRRC) Department of Radiology, West China Hospital Sichuan University, Chengdu, 610041 China

**Keywords:** Obsessive-compulsive disorder, Drug-naive, Resting-state fMRI, Fractional amplitude of low-frequency fluctuation, Multivariate classification, Support vector machine

## Abstract

**Background:**

Previous resting-state functional magnetic resonance imaging (rs-fMRI) studies have revealed intrinsic regional activity alterations in obsessive-compulsive disorder (OCD), but those results were based on group analyses, which limits their applicability to clinical diagnosis and treatment at the level of the individual.

**Methods:**

We examined fractional amplitude low-frequency fluctuation (fALFF) and applied support vector machine (SVM) to discriminate OCD patients from healthy controls on the basis of rs-fMRI data. Values of fALFF, calculated from 68 drug-naive OCD patients and 68 demographically matched healthy controls, served as input features for the classification procedure.

**Results:**

The classifier achieved 72% accuracy (*p* ≤ 0.001). This discrimination was based on regions that included the left superior temporal gyrus, the right middle temporal gyrus, the left supramarginal gyrus and the superior parietal lobule.

**Conclusions:**

These results indicate that OCD-related abnormalities in temporal and parietal lobe activation have predictive power for group membership; furthermore, the findings suggest that machine learning techniques can be used to aid in the identification of individuals with OCD in clinical diagnosis.

## Background

Obsessive-compulsive disorder (OCD) is a chronic psychiatric disorder characterized by the presence of recurrent and persistent thoughts, urges or images, and repetitive behaviors, with a lifetime prevalence of 2–3% and a 12-month prevalence of up to 1% [1–4]. This disease is one of the top 10 causes worldwide of years lived with disability, indicating its considerable severity and the burden it imposes [5].

Resting-state functional magnetic resonance imaging (rs-fMRI) provides an effective and noninvasive approach to assess neural activation and connectivity between regions. The amplitude of low-frequency fluctuation (ALFF) of the blood oxygenation level-dependent (BOLD) signal is considered a physiologically meaningful measure that detects spontaneous regional brain activity with high sensitivity and specificity in rs-fMRI [6]; altered activation has been consistently identified in several brain regions in OCD, including increased ALFF in the orbitofrontal cortex (OFC) and anterior cingulate cortex (ACC), along with decreased ALFF in the parietal cortex and cerebellum [7, 8].

However, these abnormal patterns of neural activation were identified by conventional univariate analysis in which ALFF was used to compare brain activity between a group of OCD patients and a healthy control group to identify regions with significant differences. While this type of statistical comparison can help localize regional differences that occur as a function of OCD, it cannot generally differentiate between OCD patients and healthy controls individually, because not all such group differences are guaranteed to be predictive, and there might be significant overlap between the two distributions of the pertinent metric. Moreover, traditional univariate approaches to functional magnetic resonance imaging (fMRI) analysis may overlook multivariate patterns in data [9, 10]. Recently, these univariate analyses have been complemented by the use of the multivariate pattern analyses (MVPA), in particular machine learning-based approaches, it not only learn discriminative rules from an exemplar dataset and automatically determine the group membership of novel data points but also extract spatial and/or temporal patterns from neuroimaging data [9, 11]. Attempts have been made to apply machine learning approaches to rs-fMRI data on various psychiatric disorders, including major depressive disorder [12], schizophrenia [13], mild cognitive impairment, and Alzheimer’s disease [14]. The most commonly used pattern recognition method in neuroimaging literature is support vector machine(SVM)- an algorithm uses a well-defined dataset to create decision function or “hyperplane” which can best distinguish between categories, and then the produced decision function or hyperplane will be used to predict which predefined group a new observation belongs to. Evidence of comparison studies among multivariate pattern recognition methods showed that SVM helps weigh down the effect of noisy features that are highly correlated with each other when there are a large number of features [9].

OCD is currently diagnosed on the basis of a subjective clinical interview and scale evaluation, which always leads to diagnostic inconsistency among psychiatrists, cultures, and districts [15]. Thus, researchers attempting to combine neuroimaging data with SVM techniques in recent years have found that this approach has the potential to differentiate OCD patients from healthy subjects. Classification algorithms have been applied to diffusion tensor imaging (DTI) [16], structural magnetic resonance imaging [17, 18] and task fMRI [19] with the goal of distinguishing OCD patients from healthy controls and achieved relatively satisfactory findings. Furthermore, a comparison study showed SVM achieve higher accuracy than Gaussian process classifier (GPC) using white matter features [17]. However, those previous studies included patients who were taking medication at the time of acquiring neuroimaging data; medication would affect the intrinsic patterns of neural activity and might compromise the accuracy of the classifier.

To our knowledge, no study has yet utilized SVM classification with fractional ALFF (fALFF) – an improved approach to detect spontaneous regional brain activity with higher sensitivity and specificity than ALFF – for rs-fMRI in drug-naive OCD patients to identify disease characteristics and discriminate drug-naive patients from healthy controls [20]. Characterizing useful biomarkers and developing effective diagnostic models will benefit clinical diagnosis by using distinguishing features to identify potential novel treatment targets. Thus, the aims of our study were as follows: (1) to discriminate OCD patients from healthy controls using fALFF through a machine learning approach aided by SVM; and (2) to investigate the regions of the most important discriminative features and contribute to classification discrimination.

## Methods

### Participants

According to the previous OCD classification studies, supposed expected specificity = 0.8, expected sensitivity = 0.8, δ = 0.1, α = 0.05 (two-side), the number of each group we need in the study was 63. In our study, we enrolled 68 drug-naive OCD patients and 68 sex-, age-, and education-matched healthy control participants were enrolled from 2012 to 2015 under protocols approved by the Ethics Committee of West China Hospital, Sichuan University. All participants were of Chinese Han nationality and were right handed. All provided written informed consent. OCD patients were recruited from the clinic of the Mental Health Center at West China Hospital, Sichuan University. Potential participants were interviewed and scanned using the Structured Clinical Interview for DSM-IV Axis I Disorders (SCID) and diagnosed by two experienced psychiatrists (X. Yang and Y. Yang). Participants were excluded if they had any of the following characteristics or conditions: (1) age under 18 years or over 60 years; (2) any psychiatric comorbidity identified using the SCID; (3) any history of major physical illness, cardiovascular disease, or neurological disorders; (4) any history of continuous psychotherapy; and (5) pregnancy. The Yale-Brown Obsessive Compulsive Scale (Y-BOCS) was used to rate the severity of OCD symptoms. Healthy control subjects were recruited using poster advertisements and screened using the SCID (non-patient edition) by the same psychiatrists; subjects with any psychiatric or neurological illness, a family history of psychiatric illness, or any history of continuous psychotherapy were excluded.

### Data acquisition and preprocessing

Resting-state fMRI data were collected with a 3 T MRI system (EXCITE, General Electric, Milwaukee, WI) equipped with an 8-channel phase array head coil. The resting-state functional images were obtained via a gradient-echo echo-planar imaging (EPI) sequence (TR = 2000 ms, echo time = 30 ms, flip angle = 90°, slice thickness/gap = 5/0 mm, field of view = 240 × 240 mm, Matrix = 128 × 128, yielding an in-plane voxel dimension of 1.875 × 1.875 mm, 30 axial slices, 200 volumes in each run, scan time = 8 min). During the MR examination, participants were instructed to relax their minds and keep their eyes closed but not to fall asleep. Foam padding and earplugs were used to reduce head motion and scanner noise.

Resting-state functional images were preprocessed using the software Data Processing Assistant for rs-fMRI (DPARSF), version 2.3 (State Key Laboratory of Cognitive Neuroscience and Learning, Beijing Normal University) [21] on the MATLAB platform. The first 10 images were removed in consideration of magnetization saturation effects and participants’ adaptation to the environment; the remaining 190 EPI images were subjected to slice-timing correction, realigned to the first image in the first series, and subsequently unwrapped to correct for susceptibility-by-movement interactions. We obtained the time course of head motion by estimating the translation in each direction and the rotation on each axis for each of the 190 consecutive volumes. Each participant’s head movement measured less than 1.5 mm in maximum displacement and less than 1.5° in angular motion about each axis. After being realigned, all of the data were normalized to the Montreal Neurological Institute (MNI) template, resampled to 3 × 3 × 3 mm in Statistical Parametric Mapping version 8 (SPM8), and smoothed with 8 mm full-width at half-maximum Gaussian kernel and removed linear trend. Subsequently, nuisance covariates, including head motion parameters, global mean signal intensity, white matter, and cerebrospinal fluid signal intensity were regressed out. A whole-brain mask was created by removing the non-brain tissue in the anatomical images using the MRIcro software (http://www.mricro.com) [6], voxels within the mask were further analyzed.

### Voxel-wise fALFF analysis

Using the REST (http://www.restfmri.net/forum, version 1.8) software, we performed fALFF based on the procedure developed by Zou [20] after preprocessing. The time series were transformed into the frequency domain to obtain the power spectrum. The square root was calculated at each frequency of the power spectrum, and the mean square root across the low frequency range (0.01–0.08HZ) was obtained; this mean was defined as ALFF [6]. The fALFF was calculated as the ratio of the power in the low frequency range to the power across the entire frequency range (0–0.25HZ). Finally, the resulting spatial fALFFs maps were then normalized with each voxel divided by the whole-brain fALFFs mean, providing ‘mfALFF’ spatial maps.

### SVM analysis

As a supervised machine learning algorithm, an SVM performs pattern classification by finding a decision function or boundary that enables classification [10]. The SVM classifier is provided with examples in the form <x,c>, where x presents a spatial pattern (e.g., fALFF map) and c is the class label; using these examples, it is trained to find the hyperplane that best separates the input space. During the training phase, the SVM finds the hyperplane that best separates the examples in the input space according to their group labels (e.g., OCD vs HCS). After the hyperplane is determined from the training data, it can be used to predict the group membership of a new test example. In this study, SVM was applied using the PROBID (Pattern Recognition of Brain Image Data) software package (https://www.kcl.ac.uk/ioppn/depts/neuroimaging/research/imaginganalysis/Software/PROBID.aspx)as some previous studies [16, 17, 22, 23] to investigate classification accuracy of rs-fMRI images using voxel-wise fALFFs as features. A linear kernel SVM was adopted to reduce the risk of over-fitting the data, and the weight vector was extracted as an image (i.e., the SVM discrimination map). The PROBID allows a linear kernel matrix to be pre-computed and supplied to the classifier. This approach increases computational efficiency significantly and permits whole-brain classification without requiring explicit dimensionality reduction [24]. The linear kernel only has one parameter(C) that controls the trade-off between having zero training errors and allowing misclassifications. This is fixed at C = 1 for all cases (default value).

We used ‘leave-one-out’ cross-validation (LOOCV) to validate the performance of the proposed approach. A single sample from each group was designated as a test sample, while the remaining samples were used to train the classifier, and then the subject pair excluded was used to test the ability of the classifier to reliably distinguish between groups (e.g., OCD vs. HCS). This procedure was repeated for each subject pair to estimate the overall accuracy of the SVM [9, 25]. The statistical significance of the overall classification accuracy was determined by permutation testing, which consisted of repeating the classification process 1000 times with a different random permutation of the training group labels and counting the number of permutations having higher sensitivity and specificity than the true labels. Then the number was used to derive a *P* value [22, 26]. The receiver operating characteristic (ROC) curve was plotted to show classifier performance; classification accuracy describes the proportion of correct predictions at a particular decision threshold.

### Discrimination maps

Since the SVM classifiers are multivariate techniques and discrimination is based on the brain-wide pattern instead of patterns in individual regions, all voxels contributed to the classification, and local inferences should not be made. We selected the peak of the SVM weight vector for each classifier, setting the threshold to 30% of the maximum weight vector value, an approach that is consistent with previous studies [16, 26, 27]. This threshold nearly eliminates noise components, enabling a better visualization of the most discriminating regions [26].

## Results

### Demographics and clinical characteristics

There were no significant differences in gender, age, and education years between OCD patients and healthy controls. In the OCD group, the mean duration of OCD symptoms and Y-BOCS score are shown in Table 1.

**Table 1:** Demographics and clinical characteristics of drug-naive OCD patients and health controls

	OCD (range)	Controls (range)	Analysis	*P*-value
Number	68	68		
Gender				
Male	45	45	0.000	1.000
Female	23	23		
Age (years)	27.99 ± 8.19 (18~43)	27.57 ± 8.57 (18~40)	0.286	0.775
Education (years)	13.83 ± 2.72 (7~19)	13.25 ± 3.32 (8~19)	1.117	0.266
Duration of illness (years)	6.40 ± 5.20 (0.5~16)	–	–	–
Y-BOCS total Score	21.53 ± 5.38 (10~29)	–	–	–
Obsessions subscale	13.94 ± 5.22 (0~16)	–	–	–
Compulsion subscale	7.59 ± 5.56 (0~17)	–	–	–

*OCD* Obsessive-Compulsive Disorder, *Y-BOCS* Yale-Brown Obsessive Compulsive scale

### Classification performance

**Fig. 1:**
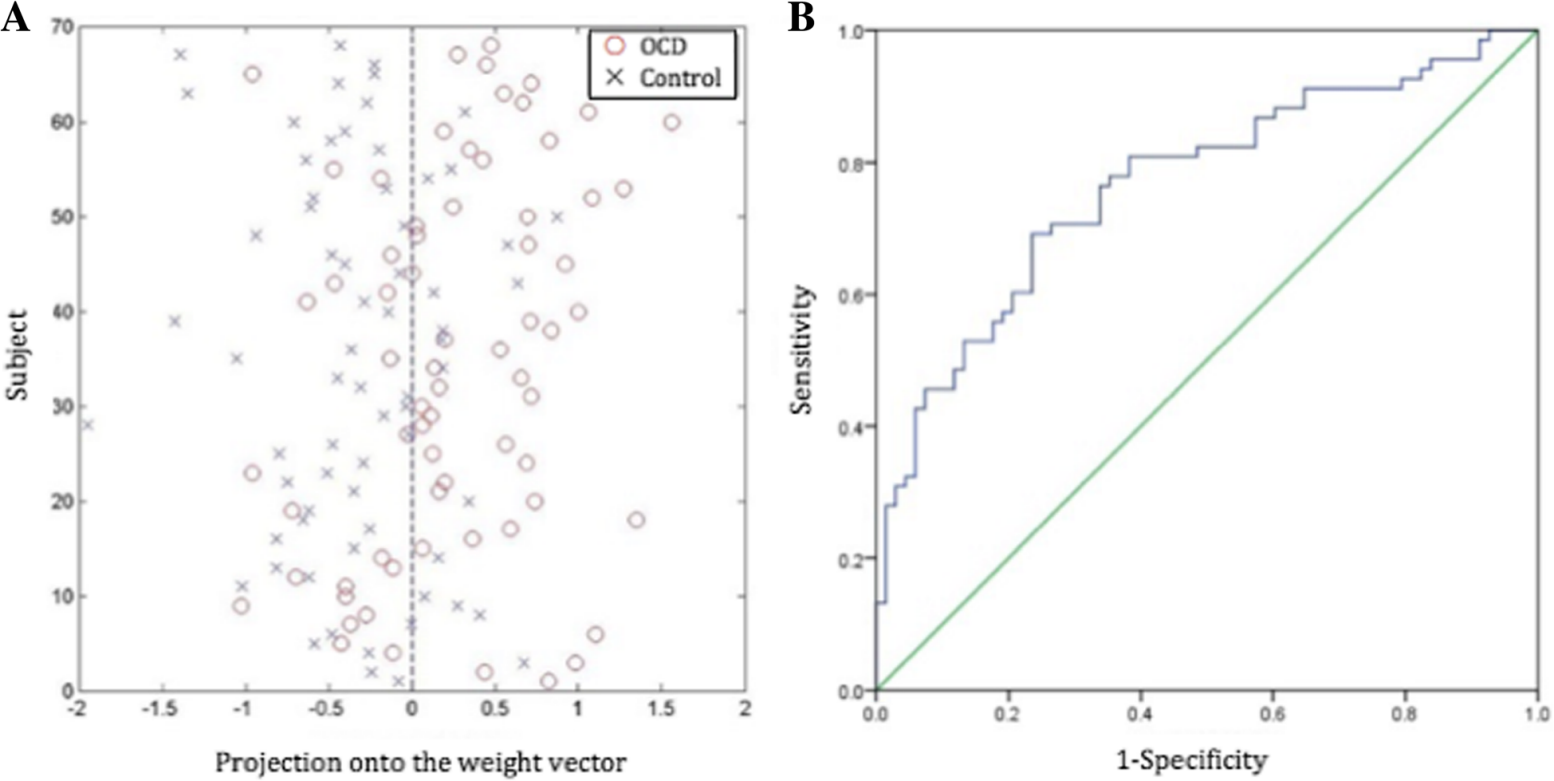
Classification plot and ROC curve of OCD patients and healthy controls

Figure 1a shows the results of the SVM classification of OCD patients and healthy controls based on the fALFF values derived from rs-fMRI data. Sensitivity (i.e., the probability that a volunteer with a clinical diagnosis of OCD was correctly assigned to the OCD category) was 68%, and specificity (i.e., the probability that a healthy control was correctly classified as such) was 76%; overall accuracy was 72% (standard error 0.051 and a 95% confidence interval of 0.687–0.847, with the ROC curve shown in Fig. 1b), and permutation tests indicated that the accuracy of classification was statistically significant at *P* < 0.001. This overall classification accuracy of the algorithm measures its ability to correctly classify an individual as either an OCD patient or a healthy control.

Classification plot (Fig. 1a) and ROC curve (Fig. 1b) for the comparison between drug-naive OCD patients and healthy controls using fALFF maps from rs-fMRI data.

### Discrimination map of OCD abnormalities

**Fig. 2:**
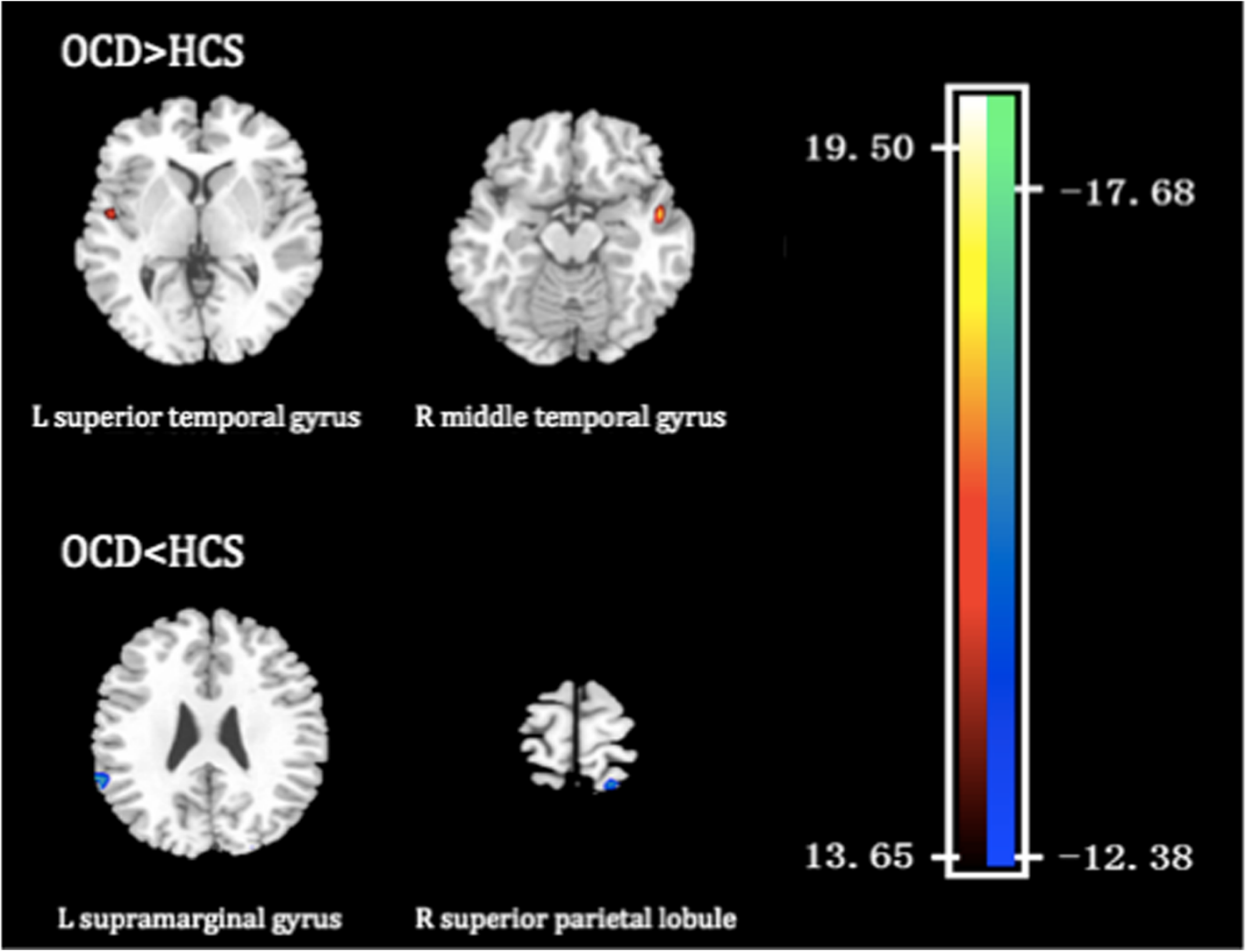
Discrimination map of OCD abnormalities

Across the brain, the regions that made the most substantial contribution to the discrimination between OCD patients and healthy controls were determined on the basis of fALFF values, which were identified by setting the threshold to ≥30% of the maximum weight vector scores. Spatial maps of the regions are described in Table 2 and shown in Fig. 2; these regions include the left superior temporal gyrus, the right middle temporal gyrus, the left supramarginal gyrus, and the superior parietal lobule.

**Table 2:** Regions contributing to discrimination between the drug-naive OCD and healthy control subjects on the basis of fALFF values

Brain regions	MNI coordinates			*Wi*
	x	y	z	
OCD > HCS				
L superior temporal gyrus	− 49	−4	4	15.16
R middle temporal gyrus	53	−1	−16	19.44
OCD < HCS				
L supramarginal gyrus	−49	−51	26	−15.91
R superior parietal lobule	20	−58	74	−15.73

The regions were identified by setting the threshold to ≥30% of the maximum weight vector, the value of which indicates the relative contribution to the classification. *OCD* obsessive-compulsive disorder, *fALFF* fractional amplitude of low-frequency fluctuation, *HCS* healthy control subjects, *L* left, *R* right, *MNI* Montreal Neurological Institute, *Wi* weight vector value.

Brain regions contributing to discrimination between the OCD and healthy control groups based on fALFF. These regions were identified by setting the threshold to ≥30% of the maximum weight vector scores. Positive weights (warm colors) indicate that the parameter values are higher in OCD patients than in healthy controls; negative weights (cool colors) indicate the opposite. The color bar represents the weight vector value (Wi) from the SVM analysis.

## Discussion

To the best of our knowledge, this study is the first to employ a machine learning approach to rs-fMRI data for clinical application in in drug-naive OCD patients. We designed an SVM method to distinguish OCD patients from healthy controls and used LOOCV to validate our model. Our study demonstrated that patients with OCD could be distinguished from healthy controls with relatively high classification accuracy using fALFF values extracted from rs-fMRI data. This classification was driven by a distributed pattern of regional abnormalities in the temporal lobe, including the left superior temporal gyrus and right middle temporal gyrus, and in the bilateral parietal lobe, including the left supramarginal gyrus and right superior parietal lobule.

A previous work achieved 84% accuracy according to LOOCV by developing a model from the DTI characteristics of 28 OCD patients and 28 healthy controls [16]. A similar method was applied using the gray matter volume (GMV) characteristics of 33 OCD patients and 33 healthy controls, and the model achieved 75.76% accuracy [17]. By contrast, our results were based on a larger dataset of rs-fMRI data from drug-naive OCD patients, which makes the classification results more stable and reliable. The main reasons for the slightly lower classification accuracy despite the larger sample size may be the different feature used for classification (i.e., resting-state regional activity vs. structural GMV and DTI) and the different medication status of subjects (i.e., drug-naive patients vs. medicated patients). Although there is increasing evidence that fALFF may be used to efficiently identify OCD in the future, in practical clinical diagnosis, more studies involve more features, and machine learning methods need to be compared to identify the information that will most improve the diagnostic accuracy of OCD.

Previous univariate analyses have shown that abnormalities of classical orbitofronto-striatal circuits cannot fully explain the cognitive defects found in OCD. Further evidence in recent studies revealed the involvement of extensive brain regions in the pathophysiology of OCD; for example, the temporal gyrus has been shown to be a critical neural substrate for OCD [28]. Previous studies using traditional univariate methods have demonstrated abnormalities in GMV in the medial temporal cortex and the precuneus, along with increased fractional anisotropy (FA) in the bilateral superior temporal region, in drug-naive OCD [29, 30]. Additionally, increased functional connectivity in the right superior temporal cortex [31] and medial temporal gyrus [32] was detected. In this study, fALFF alteration in the temporal lobe was also consistently selected as a discriminative feature, which was consistent with previous multivariate pattern analyses based on FA values and GMV [16, 17]. Some neuropsychological studies have demonstrated significant impairment of visuospatial function, which may be related to the temporal cortex, in patients with OCD [33, 34]. Consistent with previous studies, our finding revealed relatively high discriminative values for the bilateral temporal regions, supporting the notion that the temporal lobe is critically affected in OCD.

In addition, the parietal lobe, including the left supramarginal gyrus and the right superior parietal lobule, showed decreased activity. The parietal lobe is important in a variety of cognitive executive tasks involving attention, spatial perception [35, 36], planning [37], and response inhibition [38]. Deficits in attentional shifting [39], planning [40], and response inhibition [41] are evident in OCD; thus, it is conceivable that parietal lobe dysfunction could contribute to the cognitive deficits evident in OCD. Both structural and functional neuroimaging studies provide evidence to illuminate the alteration of the parietal lobe in OCD, including decreased gray matter volume in the angular and supramarginal gyri of the right parietal lobe [42], a change that is associated with attentional impairments. An rs-fMRI study also showed decreased activation in this region [7]. Additionally, after treatment and symptom improvement, activation related to the Stroop task increased [43]. Collectively, our results are in agreement with previous studies, providing further evidence for the involvement of the parietal lobe in the pathophysiology of OCD.

In summary, this study represents an important step toward the clinical diagnosis of OCD with the aid of machine learning techniques. This study does have some limitations. First, single imaging modality data and a classification approach were evaluated. Further studies will need to address these issues by introducing classification to multimodal neuroimaging data and assessing different classification methods (i.e., Gaussian Process classification, Minimum spanning tree etc.) to identify the optimal approach to discrimination. Second, the high dimensionality often induces the problem of collinearity. Although the linear kernel matrix implicated in PROBID could directly extract weight vector as an image and permits whole-brain classification without requiring explicit dimensionality reduction, the collinearity might still inevitable. Third, our research only compared OCD with HCS, other psychiatric disorders such as major depression and anxiety are not considered. Moreover, OCD patients with different dimensional symptoms could be compared to detect the pathophysiology of the symptoms. At last, the lack of follow-up limits the application of this study in predicting the treatment response of OCD. A suitable continuation of this study would be to focus on the discrimination of treatment outcomes using machine multivariate pattern recognition methods.

## Conclusions

We investigated functional abnormalities in OCD patients using a multivariate classification and explored the predictive value of fALFF in drug-naive OCD patients using an SVM framework. The SVM achieved an accuracy of 72% in LOOCV and provided good group separation. In our study, the fALFF values in the left superior temporal gyrus, the right middle temporal gyrus, the left supramarginal gyrus and the superior parietal lobule were identified as discriminative features distinguishing OCD patients from healthy controls. Our study not only identified functional biomarkers of drug-naive OCD patients but also revealed their discriminative power in distinguishing patients from controls. This study highlights the potential of machine learning approaches to aid in the clinical diagnosis of OCD.

## Data Availability

The data will be available from the author upon reasonable request. The dataset will not be publicly available because it contains information that could compromise the participants’ privacy.

## Abbreviations

ACC: Anterior cingulate cortex
ALFF: Amplitude of low-frequency fluctuation
AUC: Area under the receiver operating characteristic curve
BOLD: Blood oxygenation level-dependent
DPARSF: Data processing assistant for resting-state functional magnetic resonance imaging
DTI: Diffusion tensor imaging
EPI: Echo-planar imaging
fALFF: Fractional amplitude of low-frequency fluctuation
fMRI: Functional magnetic resonance imaging
GMV: Gray matter volume
LOOCV: Leave-one-out cross-validation
MNI: Montreal Neurological Institute
OCD: Obsessive-compulsive disorder
OFC: Orbitofrontal cortex
PROBID: Pattern Recognition of Brain Image Data
ROC: Receiver operating characteristic
rs-fMRI: Resting-state functional magnetic resonance imaging
SCID: Structured Clinical Interview for DSM-IV Axis I Disorders
SVM: Support vector machine
Wi: Weight vector value
Y-BOCS: Yale-Brown Obsessive Compulsive Scale
